# The Influence of Oil leaking rate and Ocean Current Velocity on the Migration and Diffusion of Underwater Oil Spill

**DOI:** 10.1038/s41598-020-66046-1

**Published:** 2020-06-08

**Authors:** Hong Ji, Manlin Xu, Weiqiu Huang, Ke Yang

**Affiliations:** 1 Jiangsu Key Laboratory of Oil & Gas Storage and Transportation Technology, Changzhou University, Changzhou, 213016 Jiangsu China; 2 School of Petroleum Engineering, Changzhou University, Changzhou, 213016 Jiangsu China; 3 School of Environment and Safety Engineering, Changzhou University, Changzhou, 213164 Jiangsu China

**Keywords:** Environmental impact, Sustainability

## Abstract

Severe environmental pollution and huge economic losses would be caused by submarine oil spill with the increasing development of petroleum energy in sea. In order to predict the law of migration of oil spill from submarine pipelines accurately, the volume of fluid (VOF) model and realizable *k-ε* turbulence model were employed to establish numerical simulation of submarine oil spill, and the experiments were used to verify the feasibility of the numerical models. Different oil leaking rate and ocean velocity were simulated in the study. The simulation results indicated that comparing with oil leaking rate (set vertical migration velocity, *U*_o_), current velocity (set horizontal migration velocity, *U*_w_) has a greater influence on the migration of the oil spilling; the actual vertical migration velocity (*U*_o1_), actual horizontal migration velocity (*U*_w1_) and *R*_1_ (the ratio of *U*_o1_ and *U*_w1_) are positively correlated with *R* (the ratio of *U*_o_ and *U*_w_), and they both fluctuate within a small range no matter how large *R* is; when 20 ≤ *R* ≤ 150, *R*_1_ fits a linear fit curve with curve as *R*_1_ = 0.66932 + 0.00215 *R*, which can provide a theoretical reference to the recovery system of underwater pipeline oil spilling emergency.

## Introduction

With the gradual depletion of land resources, people have turned their eyes to the ocean. Submarine pipelines are the lifelines of offshore oil and gas fields. However, submarine pipelines are in a harsh environment for a long time and normally damaged under various unforeseen risks^1^, which could cause oil spills^2^. In the event of oil spill, it will cause huge economic losses and lead to a series of adverse social effects. Therefore, accurate prediction of the diffusion pattern and behavior of oil spills is important to the risk assessment of oil spills, emergency response and control of the pollutants.

In recent decades, more than 50 models have been developed to predict the behavior and process of oil spills^3–5^. Hirst^6^ numerically investigated 2D and 3D models of buoyant jets of oil spill, and verified correctness of them by comparing them with the experimental results under currents. Mcdougall^7^, Milgram^8^ and Fanneløp, *et al*.^9^ established well oil spill models without considering the action of currents. Bemporad^10^ simulated the buoyant jet trajectories of a circular hole in stratified flow. Yapa *et al*.^4^ established an underwater oil spill model for shallow water environments. Johansen^11^, Yapa and Zheng^4,12^ established the deep sea oil spill models DeepBlow and CDOG respectively, which both have been well applied in the emergency treatment and prediction process of submarine oil spill accidents. And after that, these two models were revised and improved by other scholars, and the underwater oil spill was simulated and predicted successfully^13–15^. Ben-Mansour *et al*.^16^ established a 3D turbulence model to simulate small-diameter underwater leakage under realistic velocities and pressures, which showed significant features in the pressure and pressure gradient variations along the pipeline. Reed *et al*.^17^ established a POSCOEM model including the Release Module and the Near Field Module to estimate the leakage of the submarine pipeline. Certain scholars in China have also begun to study the numerical simulation of underwater oil spills actively. Wang *et al*.^5^ simulated the oil spill and sea surface drift of submarine pipelines based on POM and FVCOM hydrodynamic models. Liao *et al*.^18^ established an oil spill model to investigate the dynamic behavior of oil and gas under different working conditions based on the Lagrangian integral method. Chen *et al*.^19^ simulated the underwater oil spill trajectory based on Lagrangian integral method and particle tracking method. Lu *et al*.^20^ used the VOF model to perform the leakage trajectory and diffusion of oil under effects of different factors. Li *et al*.^21^ analyzed the oil and gas leakage process at different leakage locations of underwater separators of ultra-deep water. Li *et al*.^22^ established a 3D drift prediction model for semi-submersible oil and sinking oil, and developed a GIS-based Bohai sea semi-submersible bottom oil risk management information system, which realized the functions of oil drift prediction and backflow calculation of oil spill trajectory.

There are many factors affecting the spread and migration trajectory of spilled oil, including oil leaking rate, ocean current velocity, oil density, leak diameter, ambient temperature and water depth, etc. Lu *et al*.^23^ determined the influence of different factors on the submarine oil spill based on sensitivity analysis of the influencing factors. Zhu *et al*.^24–26^ investigated the underwater spread and surface drift of oil spilled from a submarine pipeline under the combined action of wave and current. Yang *et al*.^27^ established an underwater oil spill model, and verified the correctness of the model through experiments. The factors such as oil leaking rate, leak diameter, ocean current velocity and oil density were studied the impact of the migration pattern. Furthermore, some researches refer to a vertical jet introduced into a free-surface water tank in a current environment^28–31^.

When the spilled oil migrates to the free surface, it would drift away or evaporate into the atmosphere under the influence of various factors, such as wind, waves, oil area and oil thickness. As a significant factor, the oil thickness has a very close relationship with the drift range and evaporation rate of spilled oil on the free surface, which mainly depends on the density and viscosity of spilled oil. The MODIS satellite visual-spectrum broadband data^32^, and synergic use of optical remote-sensing and numerical modelling can both detect and characterize marine oil slicks^33^. And the later method has also been proved correct in Literature^34^.

**Figure 1:**
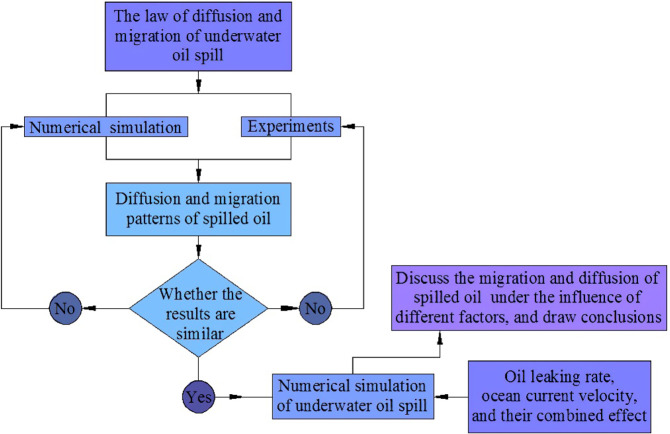
Research method and steps.

According to previous studies, the oil leaking rate and ocean current velocity are respectively two factors that have a greater impact on migration pattern of submarine oil spill. Most of the existing investigations are carried out on the influence of various single factors on migration and diffusion of underwater oil spill, and there is a lack of investigation of influence of multiple factors on it. Moreover, most of the literatures have investigated the correlation between factors and migration velocity (positive or negative), and there is a lack of experimental or numerical data did not do more quantitative analysis between factors and oil migration and diffusion parameters. Therefore, the migration pattern of submarine oil spill under the combined action of oil leaking rate and current velocity will be discussed in this study. The research methods and steps of this paper are shown in Fig. 1.

## Mathematical model and numerical approach

### Governing equations

The underwater diffusion process and surface drifting of oil spill both follows three laws of mass conservation, momentum conservation and energy conservation. Oil and water are set as incompressible fluids. The turbulence equation uses the realizable *k-ε* model, and the VOF model is used to obtain the volume fraction equation and the momentum equation. Conservation equations are as follows:

The RANS (Rey-nolds-Averaged-Navier-Stokes) equations are used to describe liquid flow, which including continuity equations and momentum conservation equations as follows^35^:

1 $$\frac{\partial {u}_{i}}{\partial {x}_{i}}=0$$

2 $$\frac{\partial {u}_{i}}{\partial t}+\frac{\partial {u}_{i}{u}_{j}}{\partial {x}_{j}}=-\frac{\partial \overline{p}}{\rho \partial {x}_{i}}+\upsilon {\nabla }^{2}{u}_{i}-\frac{\partial {x{\prime} }_{i}{x{\prime} }_{j}}{\partial {x}_{j}}+{g}_{i}$$

where: *u*_*i*_ and *u*_*j*_ indicate instantaneous velocity components in *i* and *j* directions, respectively; *x*_*i*_ indicates the spatial coordinate in *i* direction; *g*_*i*_ is the gravitational acceleration in *i* direction; *t* indicates the time; *p* indicates the pressure; *ρ* and *v* represent the density and kinematic viscosity, respectively.

The VOF method is a solution to the fluid volume fraction equation based on a mixed phase momentum equation^36,37^. In this study, *F*_*w*_ and *F*_*o*_ are used to represent the fluid volume fraction in the water region and the oil region, respectively. The physical meaning of the *F* function is the fraction of the liquid phase volume of a unit. The liquid volume functions *F*_*w*_ and *F*_*o*_ are written as follows:

3 $${F}_{w}=\frac{{V}_{w}}{{V}_{c}}$$

4 $${{F}}_{{o}}{=}\frac{{V}_{o}}{{V}_{c}}$$

Where: *F*_*w*_ and *F*_*o*_ are volumetric functions of water and oil, respectively; *V*_*c*_, *V*_*w*_ and *V*_*o*_ are the volume of one unit volume, one unit of water and oil, respectively. And the subscript *w* and *o* indicate water and oil, respectively.

Fractional function of two-dimensional transport equations:

5 $$\frac{\partial {F}_{w}}{\partial t}+\frac{\partial u{F}_{w}}{\partial x}+\frac{\partial v{F}_{w}}{\partial y}=0$$

6 $$\frac{\partial {F}_{o}}{\partial t}+\frac{\partial u{F}_{o}}{\partial x}+\frac{\partial v{F}_{o}}{\partial y}=0$$

The density and kinematic viscosity are written as follows:

7 $$\rho =(1-{F}_{o}){\rho }_{w}+{F}_{o}{\rho }_{o}$$

8 $$v=(1-{F}_{o}){v}_{w}+{F}_{o}{v}_{o}$$

Realizable *k-ε* turbulence model^38–40^ including two equations for turbulent kinetic energy and turbulent kinetic energy dissipation rate is written as:

9 $$\rho \frac{\partial k}{\partial t}+\rho {u}_{i}\frac{\partial k}{\partial {x}_{i}}=\frac{\partial }{\partial {x}_{j}}\left[\left(\mu +\frac{{\mu }_{t}}{{\sigma }_{k}}\right)\frac{\partial k}{\partial {x}_{j}}\right]+{G}_{{\rm{k}}}+{G}_{b}-\rho \varepsilon $$

10 $$\rho \frac{\partial \varepsilon }{\partial t}+\rho {u}_{i}\frac{\partial \varepsilon }{\partial {x}_{i}}=\frac{\partial }{\partial {x}_{j}}\left[\left(\mu +\frac{{\mu }_{t}}{{\sigma }_{\varepsilon }}\right)\frac{\partial \varepsilon }{\partial {x}_{j}}\right]+\rho {C}_{1}S\varepsilon -\rho {C}_{2}\frac{{\varepsilon }^{2}}{k+\sqrt{\upsilon \varepsilon }}+{C}_{1}\varepsilon (1-{C}_{\varepsilon })\frac{\varepsilon }{{\rm{k}}}{G}_{{\rm{b}}}$$

where: $${\mu }_{t}=\rho {C}_{\mu }\frac{{k}^{2}}{\varepsilon }$$, $${G}_{k}=-\rho {u{\prime} }_{i}{u{\prime} }_{j}\frac{\partial {u}_{j}}{\partial {x}_{j}}$$, $${G}_{b}=-\,g\frac{{\mu }_{t}}{{\Pr }_{t}}\frac{\partial \rho }{\rho \partial {x}_{i}}$$, $${C}_{1}=\,{\rm{\max }}\left(0.43,\frac{\eta }{\eta +5}\right)$$, $$\eta =S\frac{k}{\varepsilon }$$, $$S=\sqrt{2{S}_{ij}\cdot {S}_{ij}}$$ n, $${S}_{ij}=\frac{1}{2}\left(\frac{\partial {u}_{i}}{\partial {x}_{j}}+\frac{\partial {u}_{j}}{\partial {x}_{i}}\right)$$, *C*_1_ = 1.44, *C*_2_ = 1.92, *C*_*ε*_ = 1, *C*_*μ*_ = 0.09, *Pr*_*t*_ = 0.85, *σ*_*k*_ = 1, *σ*_*ε*_ = 1.2

In these equations: *G*_*k*_ and *G*_*b*_ represent the turbulent energy *k* caused by the average velocity gradient and buoyancy, respectively; *k* and *ε* indicate the amount of enthalpy per unit mass and the rate of turbulent flow energy dissipation, respectively; *σ*_*k*_ and *σ*_*ε*_ are turbulent prandtl numbers; *μ* and *μ*_*t*_ indicate the dynamic viscosity and turbulent viscosity, respectively.

### Computational domain and boundary conditions

A 2D flow model with a leak of 0.02 m in diameter was employed, and the whole computational domain was a rectangle with a length of 10 m and a height of 4 m. The coordinate origin was located at the lower left corner of the domain. In terms of numerical simulation, the densities of water and oil were set as 998 kg·m^−3^ and 730 kg·m^−3^, respectively. The viscosities of water and oil were set as 1.003 × 10^−3^ Pa·s and 2.4 × 10^−3^ Pa·s, respectively. As shown in Table 1, the oil leaking rate (*U*_o_) and ocean current velocity (*U*_w_) were varied from case to case.

**Table 1:** Simulation cases.

Case	Oil leaking rate/*U*_o_ (m·s^−1^)	Ocean current velocity/*U*_w_ (m·s^−1^)	*R* = *U*_o_/*U*_w_
1	4	0.1	40
2	10	0.1	100
3	15	0.1	150
4	4	0.2	20
5	10	0.2	50
6	15	0.2	75
7	10	0.5	20
8	10	1	10
9	12	0.1	120
10	12	0.5	24
11	12	1	12
12	4	0.8	5
13	10	2	5
14	6	0.3	20
15	3	0.1	30
16	6	0.2	30
17	9	0.3	30
18	8	0.2	40
19	12	0.3	40

**Figure 2:**
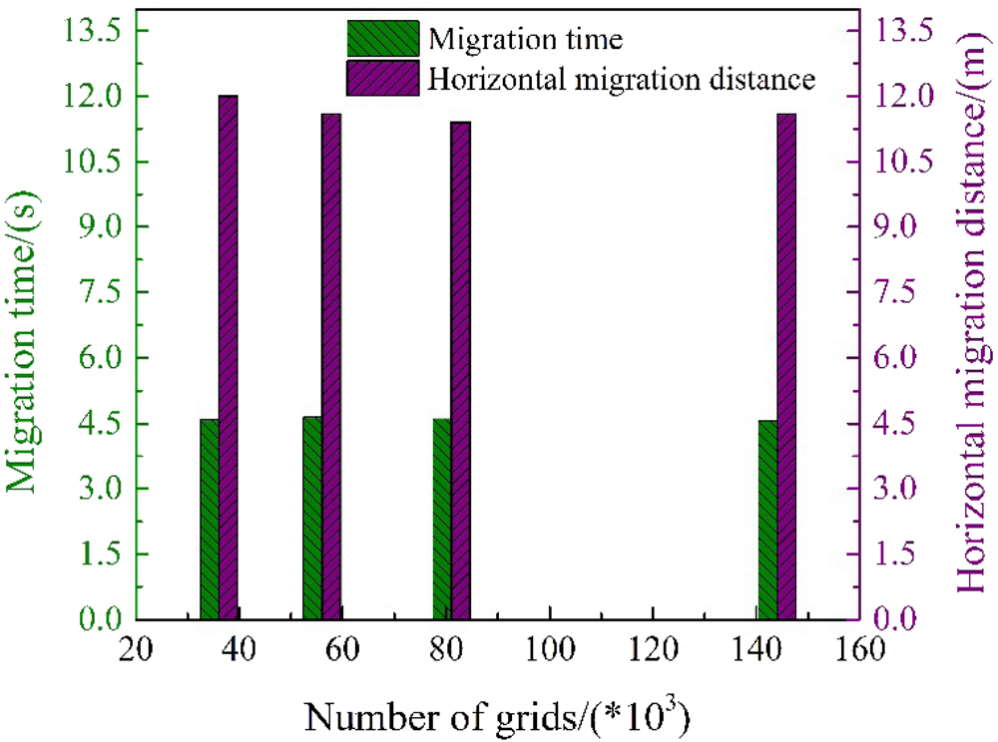
Comparison of simulation results under different numbers of grids.

In order to obtain a higher-quality simulation model, and obtain the simulation results more efficiently, the sensitivity analysis was performed on the four models with different grid numbers (36000, 56000, 81000 and 144000). In the numerical simulation of underwater oil spill, the time for the oil to migrate to the surface and the horizontal migration distance are of great significance for emergency response to underwater oil spills. Therefore, when verifying the grid sensitivity in this paper, the oil floating time to the water surface and the change of the horizontal migration distance when the migration time is 10 s under the four grid quantity models are investigated. The results are shown in Fig. 2. It can be seen that the migration time and horizontal migration distance obtained under the four grid numbers were relatively close, and the continued increase in the number of grids has little effect on the calculation results. Moreover, the calculation time of the model is positively correlated with the number of grids. In consideration of safety and calculation time, the model with the largest horizontal migration distance was selected.

### Simulation and experimental verification

**Figure 3:**
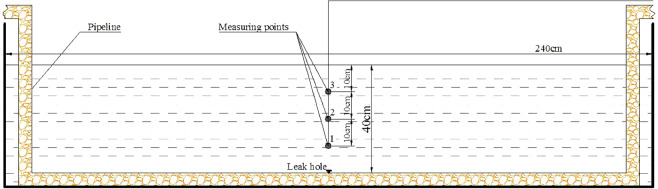
The location of the pressure measuring points.

In order to ensure the accuracy of the simulation results, experiments were carried out to judge whether the simulation model and method were feasible. The parameters of the simulation model was the same as the parameters of the experiment. In terms of the experiment and numerical simulation, the water depth was 0.4 m, the width of the water tank was 2.4 m, and the diameter of leak hole was 1.5 mm. The densities of water and oil were 998 kg·m^−3^ and 915 kg·m^−3^, respectively. The flow rate of pipeline was 44 L·min^−1^ and the oil leaking rate was 1.494 m·s^−1^. Pressure sensors and high-speed camera were applied to collect experimental data. The heights of pressure measuring points for 1 to 3 on Y axis were set at 10, 20 and 30 mm above the leak hole (Fig. 3).

**Figure 4:**
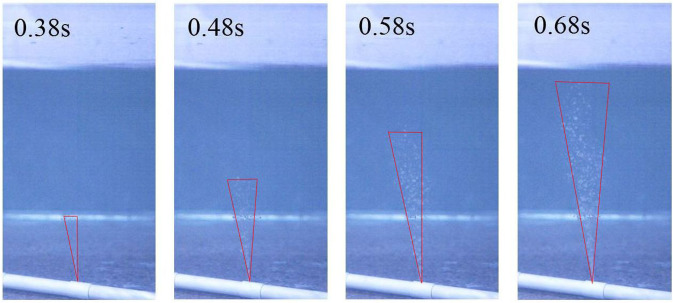
The migration and diffusion of underwater oil at the process of the experiments.

**Figure 5:**
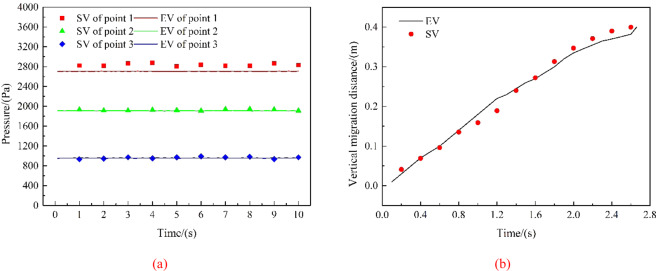
Comparison between EVs and SVs of pressure of measuring points **(a)** and vertical migration distance **(b)**.

Figure 4 presents the migration and diffusion of underwater oil at the process of the experiments. Figure 5 shows the comparison between the experimental values (EVs) and simulation values (SVs) of pressure and vertical migration distance, respectively. The SVs were generally consistent with EVs except a slight deviation between them. The error between SV and EV of the average value of the pressure at each measuring point was between 4.5% and 6.7%, and the error between SV and EV of the vertical migration velocity was 2.3%. It could be concluded that the simulation method was reasonably employed to investigate the law of diffusion and migration of submarine oil spill.

## Results and discussions

### Effect of oil leaking rate (*U*_*o*_)

**Figure 6:**
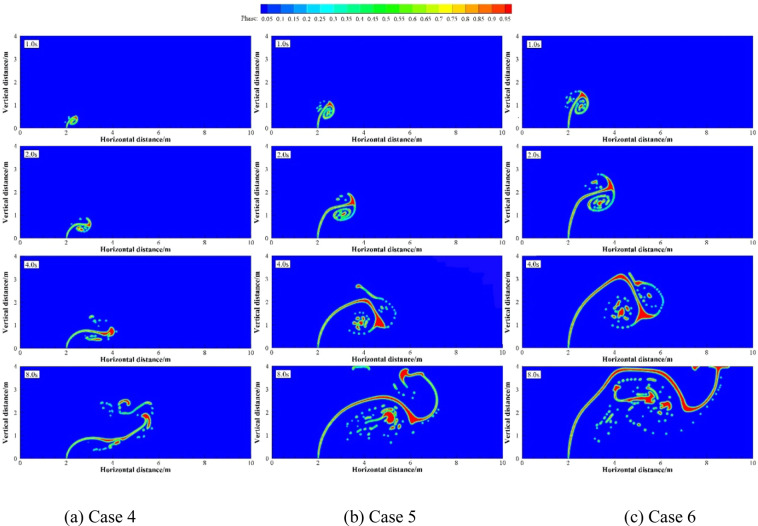
The migration and diffusion of underwater oil at different oil leaking rates when *U*_w_ = 0.1 m·s^−1^.

**Figure 7:**
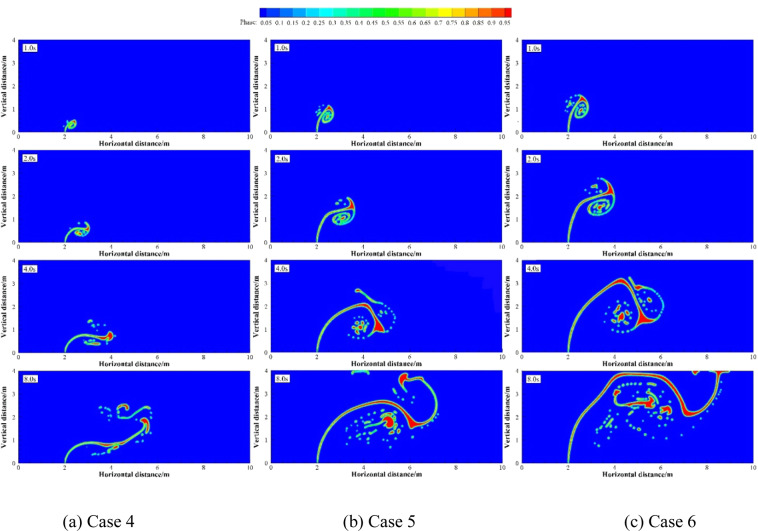
The migration and diffusion of underwater oil at different oil leaking rates when *U*_w_ = 0.2 m·s^−1^.

When studying the effects of different factors on submarine oil spills, many scholars have found that the larger the oil leaking rate, the larger the initial momentum is and the shorter the time to reach the water surface^40–43^. Numerical simulation were carried out under two groups of simulation cases (Case 1 to Case 3 as a group and Case 4 to Case 6 as a group). These two groups of cases were selected to discuss the effect of different *U*_o_ on underwater oil spill. The floating processes of oil spilling from the leak under different oil leaking rate (*U*_o_) when the current velocities are 0.1 and 0.2 m·s^−1^ are shown in Figs. 6 and 7, respectively. We assumed that the vertical migration velocity of spilled oil only under their buoyancy is the same.

Some scholars have divided the process of underwater oil spill into three stages^27,44,45^: buoyancy jet stage, buoyancy plume stage and advection diffusion stage. However, the process are divided into two stages^4,12,46^ by some other scholars: plume jet stage and buoyancy diffusion stage. The spilled vertical oil stream releases from the leak with the initial momentum, which forms the first entrainment vortexes on both sides of jet at the plume jet stage. After the initial momentum is exhausted, the dispersed oil droplets mainly migrates with the motion trail of buoyancy and ocean currents, which is the buoyancy diffusion stage.

**Figure 8:**
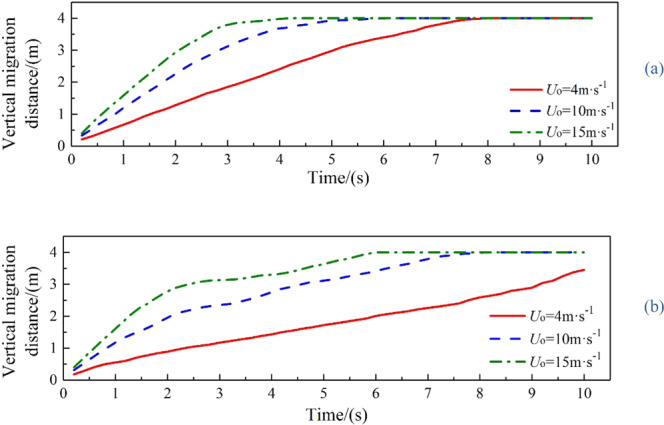
The vertical migration distance at different oil leaking rates when *U*_w_ = 0.1 m·s^−1^ (**a**) and *U*_w_ = 0.2 m·s^−1^ (**b**).

**Figure 9:**
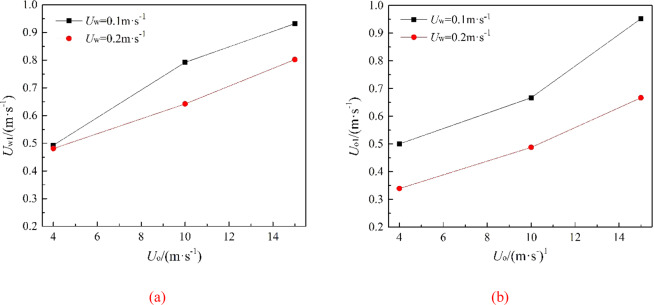
The average migration velocities of horizontal direction (**a**) and vertical direction (**b**) at different oil leaking rates when *U*_w_ = 0.1 m·s^−1^ and *U*_w_ = 0.2 m·s^−1^, respectively.

It can be seen from Figs. 8 and 9 that the larger *U*_o_ is, the larger the first entrainment vortexes are. The entrainment vortex on the left side of jet is dispersed into oil droplets of different sizes with the motion of ocean currents. However, the entrainment vortex on the right side becomes gradually larger. Then the second entrainment vortex is formed on both sides of the jet, which slows down the migration velocity of the jet. The dispersed oil droplets are accumulated at the lower right side of the jet. The larger *U*_o_ is, the more oil droplets accumulated. In this process, more and more dispersed oil droplets are separated under the control of the entrainment vortex, and move up slowly with the motion of buoyancy and ocean currents.

**Figure 10:**
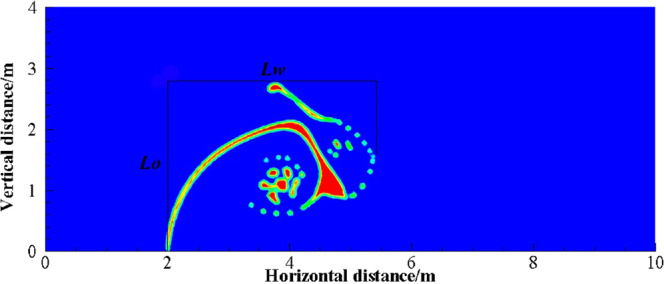
Definition of parameters of oil spill migration.

The larger *U*_o_ is, the less the initial migration pattern of oil leakage is affected by the ocean current, and the greater the change in the vertical migration velocity of the jet is. In the first stage of jet, the spilled oil in all cases migrates to the surface with different initial oil leaking rate, and the jet is less affected by the ocean current because of the initial momentum. However, in the second stage, the jet gradually loses control of the initial momentum. Spilled oil migrates along the direction of the ocean current under the influence of ocean current and buoyancy, and the jet trajectory is greatly deviated. The vertical migration velocities of jet are reduced to a same value only under the influence of buoyancy (Fig. 8). When *U*_o_ is smaller, the vertical velocity difference between two stages of jet is smaller because of the smaller initial momentum, so the vertical migration curve is smoother. It can also be seen from Fig. 9 that both the average horizontal migration velocities (*U*_w1_) and average vertical migration velocities (*U*_o1_) are linearly and positively correlated with *U*_o_. As shown in Fig. 10, we defined the migration height and migration width as the vertical migration length (*L*_o_) and horizontal migration length (*L*_w_) of spilled oil, respectively. And the ratio of *L*_o_ to migration time (*t*_o_) was *U*_o1_, and the ratio of *L*_w_ to *t*_o_ was *U*_w1_.

### Effect of ocean current velocity (*U*_*w*_)

**Figure 11:**
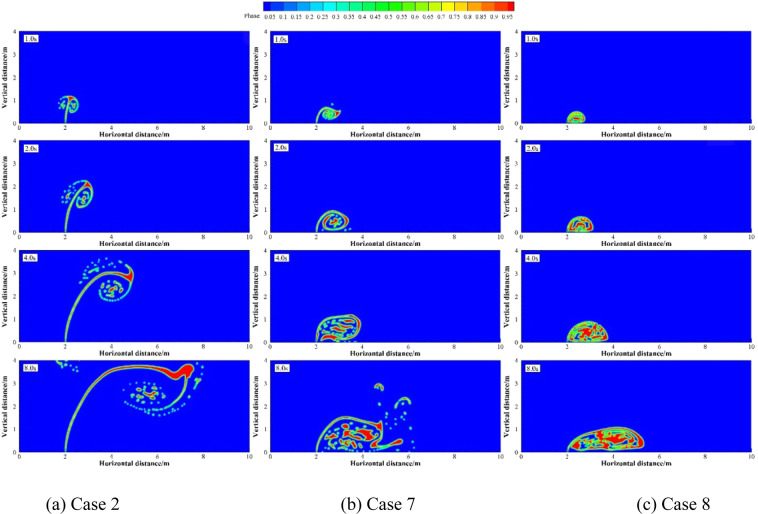
The migration and diffusion of underwater oil at different current velocities when *U*_o_ = 10 m·s^−1^.

**Figure 12:**
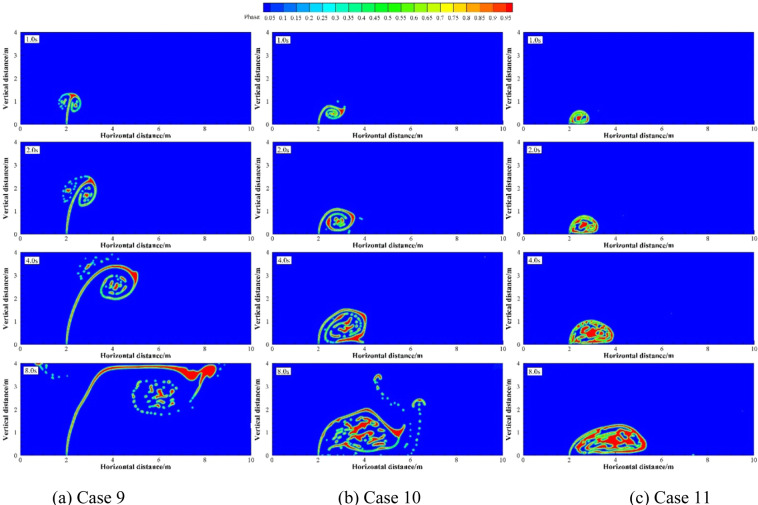
The migration and diffusion of underwater oil at different current velocities when U_o_ = 12 m·s^−1^.

Numerical simulations were carried out under two groups of simulation cases (Case 2, 7 and 8 as a group; Case 9 to 11 as a group). These two groups of cases were selected to discuss the effects of different *U*_w_ on underwater oil spill. The floating processes of oil spilling motion from the leak under different *U*_w_ when the oil leaking rates were 10 and 12 m·s^−1^ are shown in Figs. 11 and 12, respectively.

**Figure 13:**
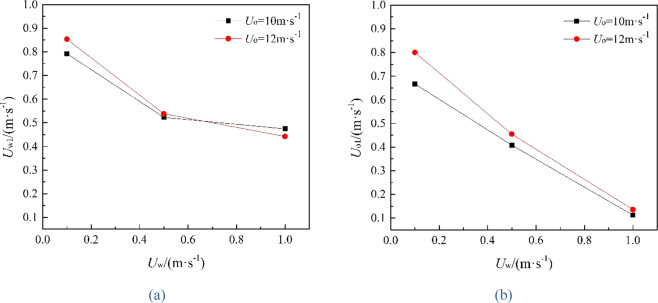
The average migration velocities of horizontal direction (**a**) and vertical direction (**b**) at different current velocities when *U*_0_ = 10 m·s^−1^ and *U*_0_ = 12 m·s^−1^, respectively.

It can be seen from the following figures that ocean current velocity also has a great influence on the migration of spilled oil. Firstly, when oil leaking rate is 10 m·s^−1^ (Fig. 11), the larger *U*_w_ is, the smaller the entrainment vortex on the left side during the first entrainment movement. When *U*_w_ = 1 m·s^−1^, the first entrainment vortex only exists on the right side. Secondly, the larger the ocean current velocity is, the more oil droplets accumulates under the control of the entrainment motion and the initial momentum; and the lower the height of the oil droplets accumulation is, the more slowly the spilled oil migrates to the surface. Finally, according to the figures, the larger *U*_w_ is, the later the second stage appears. When ocean current velocity is large enough, the influence of ocean current is obviously dominant. The oil droplets that have just overflowed are washed away by ocean current^47^, and the second entrainment motion does not occur any more (Figs. 11(c) and 12(c)). It can be seen from Fig. 13 that both *U*_w1_ and *U*_o1_ of the jet are negatively correlated with *U*_w_.

### Combined action of oil leaking rate (*U*_*o*_) and ocean current velocity (*U*_*w*_)

**Figure 14:**
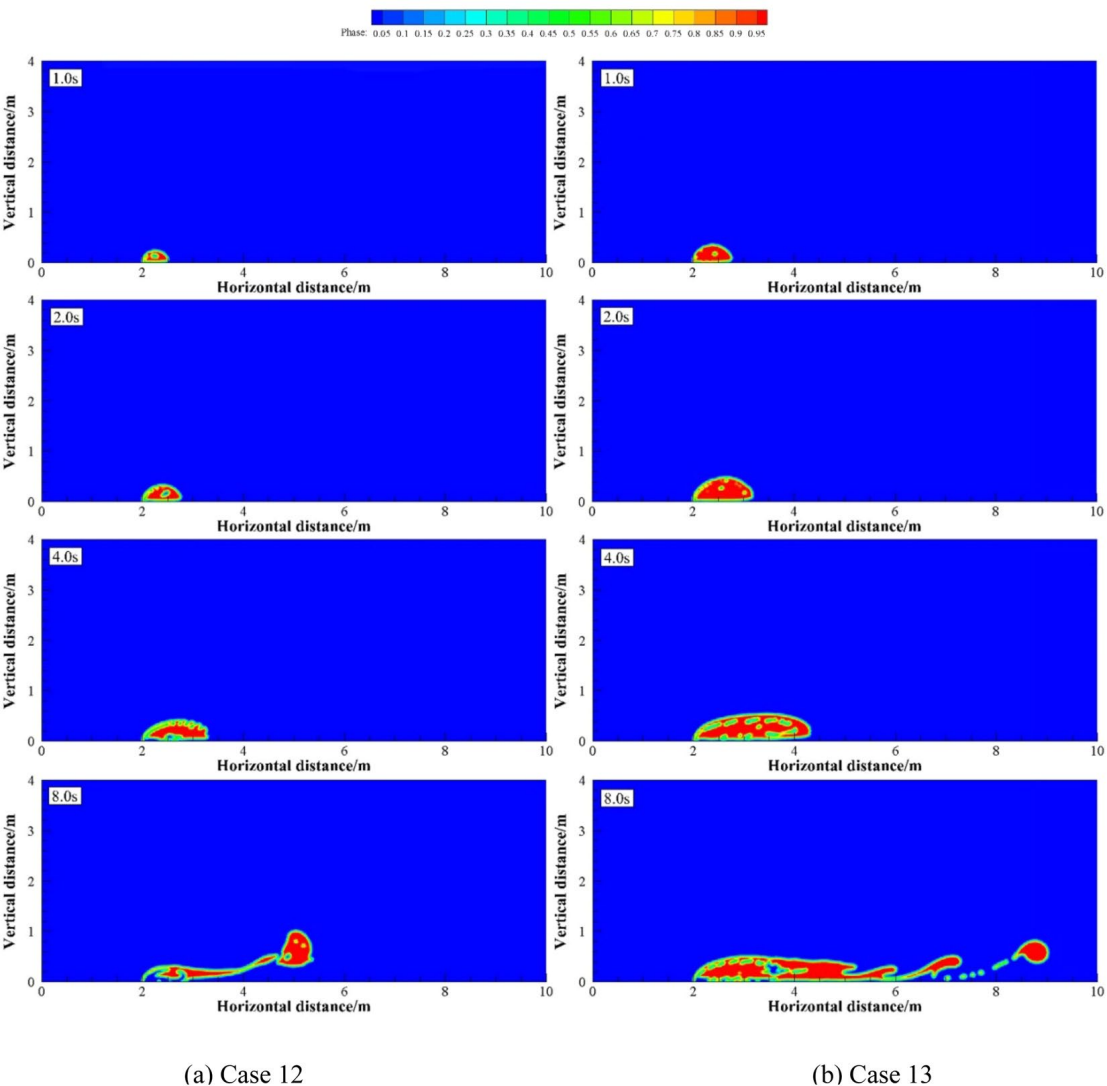
The migration and diffusion of underwater oil at different cases when *R* = 5.

**Figure 15:**
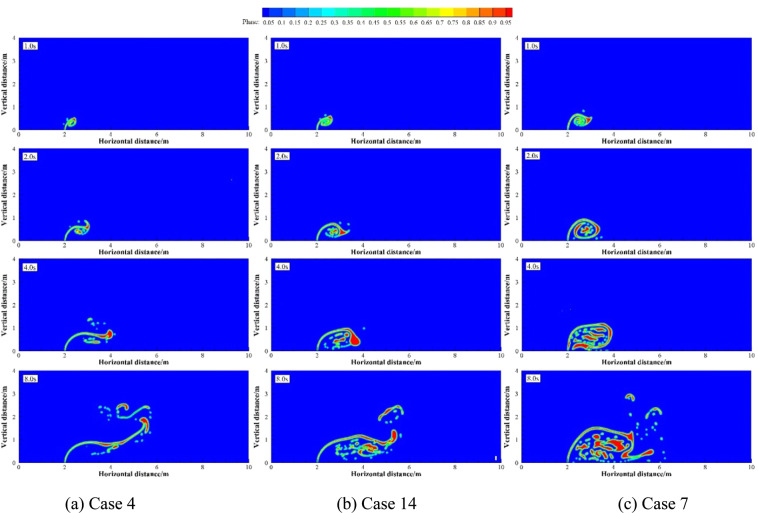
The migration and diffusion of underwater oil at different cases when *R* = 20.

**Figure 16:**
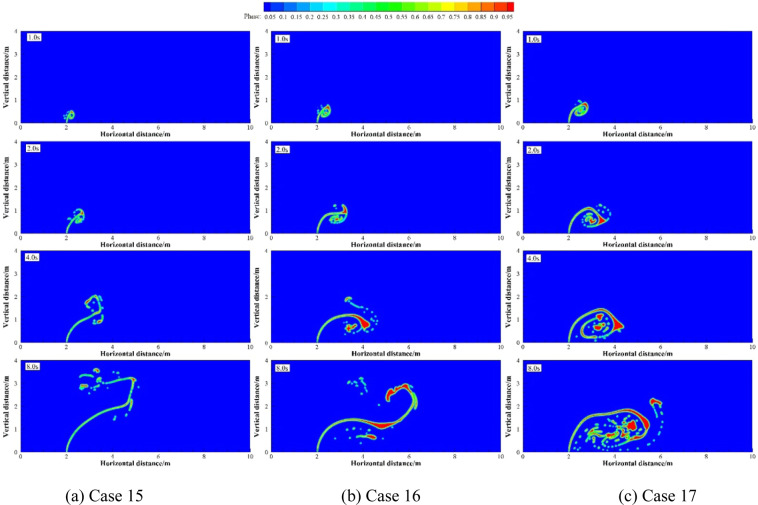
The migration and diffusion of underwater oil at different cases when *R* = 30.

**Figure 17:**
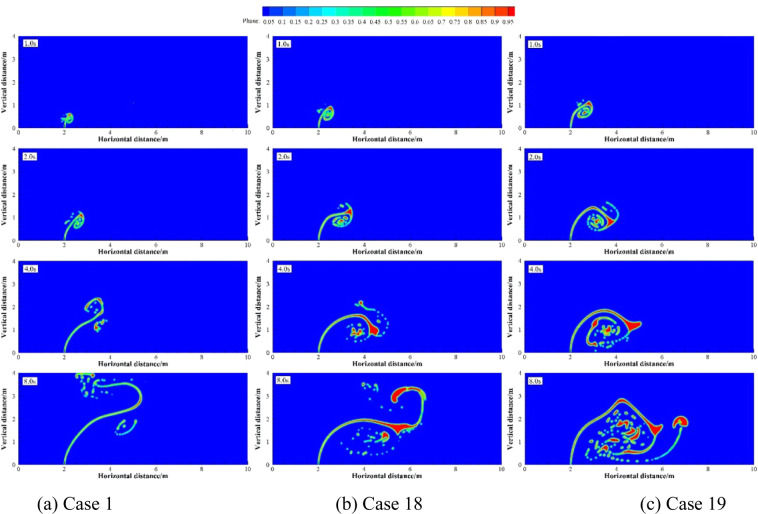
The migration and diffusion of underwater oil at different cases when *R* = 40.

Numerical simulation were carried out under four groups of simulation cases (Case 12 and 13; Case 4, 7 and 14; Case 15 to 17; Case 1, 18 and 19 as four groups, respectively). These four groups of cases were selected to discuss the effect of different *R* on underwater oil spill. The floating processes of oil spilling from the leak under different *R* are shown in Figs. 14–17.

It can be seen from the above that the increase of *U*_o_ and *U*_w_ respectively can both intensify the accumulation of oil droplets at the lower right side of the jet. The accumulation of oil under the water has a certain correlation with *U*_o_ and *U*_w_. We define that the ratio of *U*_o_ to *U*_w_ as *R*, and the ratio of *U*_o1_ to *U*_w1_ as *R*_1_.The smaller *R* is, the more dispersed oil droplets accumulate. However, according to Figs. 14–17, the same *R* cannot guarantee the migration trajectory of the spilled oil to be consistent. When *R* is the same, but both *U*_o_ and *U*_w_ increase, the migration pattern of the jet is still similar to the trajectory migration pattern when only *U*_w_ increases. However, when *R* is small enough, even if the oil leaking rate is large enough, the spilled oil rushes directly to the right of the leak hole by the ocean current at the first stage^47^ (Fig. 14). The larger *U*_o_ is, the longer the jet is affected by the initial momentum, and the later the second stage of free diffusion occurs. It can be known that, by the comparison of the two factors, the influence of *U*_w_ on the migration pattern of the oil spilling is greater than that of *U*_o_ on the jet. It can be seen that *U*_w_ is a more important reference than *U*_o_ to determine the migration and diffusion pattern and behavior of the spilled oil.

**Figure 18:**
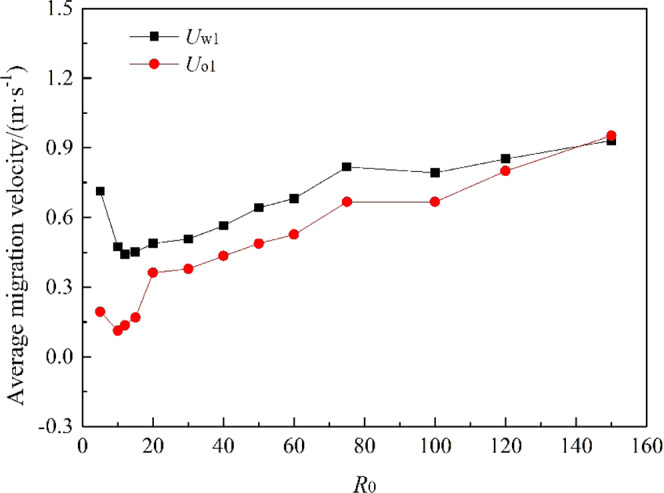
Average migration velocities at different *R*.

**Figure 19:**
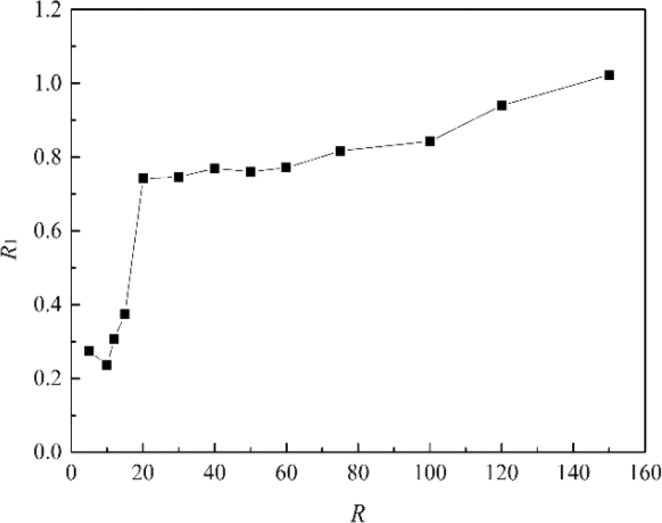
*R*_1_ (Ratio of *U*_*o*1_ and *U*_*w*1_) at different *R*.

**Figure 20:**
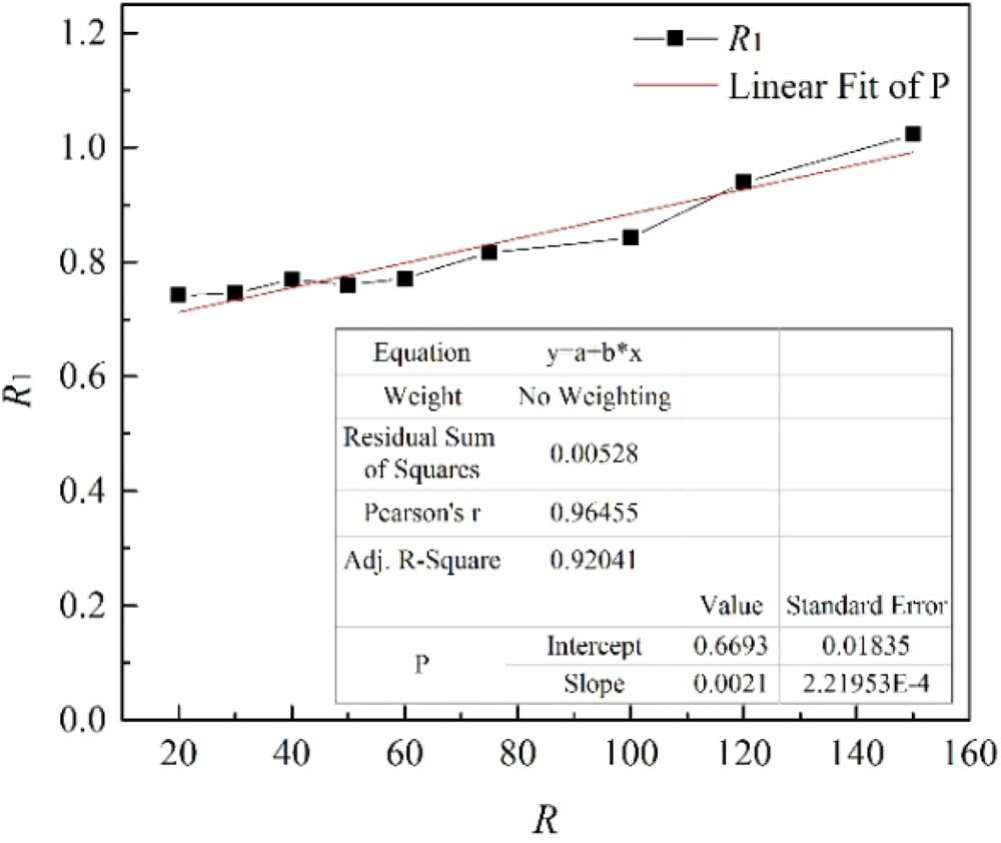
*R*_1_ fitted curve with *R* values from 20 to 150.

It can be seen from Fig. 18 that the values of *U*_o1_ and *U*_w1_ are basically the same when *R* is the same, and they are positively correlated with *R*, fluctuating within a small range. The value of *R* in this study was ranged from 5 to 150. We can obtain from Fig. 19 that *R*_1_ (*R*_1_ = *U*_o1_/*U*_w1_) is linearly positively correlated with *R*, but *R*_1_ fluctuates within a small range (0.24 to 1.02). When 20 ≤ *R* ≤ 150, *R*_1_ increases uniformly in the range of 0.75 to 1.02, and it fits a linear fit curve (as shown in Fig. 20), and the adjust R-square is 0.92041. The fitting formula is as follow:

11 $${R}_{1}=0.66932+0.00215R$$

However, under the extreme conditions of *R* < 10, this rule is not fully applicable. When *R* is small enough, the influence of the ocean current is obviously dominant, and the initial momentum is intercepted at the first stage. The spilled oil is washed away as soon as it overflows, and only when the initial momentum disappears at the second stage can it gradually migrate towards the surface.

## Conclusions

The migration and diffusion of underwater oil spills were simulated, and the effects of different *U*_o_, different *U*_w_ and the combined action of *U*_o_ and *U*_w_ on underwater oil spills were discussed. The main conclusions obtained are as follows:

1. When *U*_w_ is constant, *U*_o1_ and *U*_w1_ are linearly positively correlated with *U*_o_. The larger *U*_o_ is, the higher the initial momentum of the oil jet is, the less influential the ocean current poses on it, the wider the entrainment range is, and the more oil droplets accumulate in the vortex under the control of the entrainment movement and initial momentum. When *U*_o_ is constant, the larger *U*_w_ is, the larger the difference in the size of the first entrainment vortex on both sides of the jet is, the larger number and the lower height of oil droplets accumulate, and the more slowly the spilled oil migrates to the surface. The influence of ocean current is obviously dominant when *U*_w_ is large enough. Comparing the two factors, it can be obviously seen that the influence of *U*_w_ on the migration pattern of the oil spilling is greater than that of *U*_o_.
2. The smaller *R* is, the more dispersed oil droplets accumulate together. And when *R* is small enough, the initial momentum is controlled by the ocean current. It can only gradually float to the surface of the water under the motion of its own buoyancy and current after the initial momentum disappears.
3. The average migration velocities are positively correlated with *R*, fluctuating within a small range of 0 to 1.1 m·s^−1^. *R*_1_ (*R*_1_ = *U*_o1_/*U*_w1_) is linearly positively correlated with *R*, fluctuating within a small range of 0.2 to 1.1, no matter how large *R* is. The linear fit curve is that *R*_1_ = 0.66932 + 0.00215*R*.
